# Effect of Various Exercise Regimens on Selected Exercise-Induced Cytokines in Healthy People

**DOI:** 10.3390/ijerph18031261

**Published:** 2021-01-31

**Authors:** Remigiusz Domin, Daniela Dadej, Michał Pytka, Ariadna Zybek-Kocik, Marek Ruchała, Przemysław Guzik

**Affiliations:** 1 Department of Endocrinology, Metabolism and Internal Medicine, Poznan University of Medical Sciences, 60-355 Poznań, Poland; daniela.dadej@gmail.com (D.D.); ariadna.zybek@gmail.com (A.Z.-K.); mruchala@ump.edu.pl (M.R.); 2 Department of Cardiology-Intensive Therapy, Poznan University of Medical Sciences, 60-355 Poznań, Poland; mjpytka@gmail.com (M.P.); pguzik@ptkardio.pl (P.G.)

**Keywords:** myokines, hepatokines, exercise, myostatin, follistatin, brain-derived neurotrophic factor, decorin, fibroblast growth factor 21, interleukin 15, exercise-induced cytokines

## Abstract

Different forms of physical activity—endurance, resistance or dynamic power—stimulate cytokine release from various tissues to the bloodstream. Receptors for exercise-induced cytokines are present in muscle tissue, adipose tissue, liver, brain, bones, cardiovascular system, immune system, pancreas, and skin. They have autocrine, paracrine and endocrine activities. Many of them regulate the myocyte growth and differentiation necessary for muscle hypertrophy and myogenesis. They also modify energy homeostasis, lipid, carbohydrate, and protein metabolism, regulate inflammation and exchange information (crosstalk) between remote organs. So far, interleukin 6 and irisin have been the best studied exercise-induced cytokines. However, many more can be grouped into myokines, hepatokines and adipomyokines. This review focuses on the less known exercise-induced cytokines such as myostatin, follistatin, decorin, brain-derived neurotrophic factor, fibroblast growth factor 21 and interleukin 15, and their relation to various forms of exercise, i.e., acute vs. chronic, regular training in healthy people.

## 1. Introduction

In 2003, Pedersen et al. introduced the term “myokines’’ to describe cytokines produced and released by skeletal muscles in response to contraction [1]. Later, Seldin et al. highlighted that some of the myokines are secreted in response to exercise even in higher amounts by non-muscle tissues than by the muscles [2]. Still, the authors of the term “myokines” state that potential cytokines, to be classified as myokines, must be derived from skeletal muscle, must be secreted and must carry out a biological function in an endocrine, paracrine or autocrine mode [3,4]. Due to the main site of origin of particular cytokines and the proposed myokine classification criteria, the nomenclature among literature can be confusing. It is further complicated by the emergence of new names for groups of cytokines, such as hepatokines (e.g., follistatin, fibroblast growth factor 21) or adipomyokines (e.g., interleukin 15, irisin). So far, myokines have been considered as cytokines secreted in response to exercise. However, whether only cytokines produced by muscle cells can be called myokines is widely discussed and unresolved. Irrespective of the classification, the common feature of such cytokines is a change in their concentrations in response to exercise. For the need of this review, we assume that cytokines secreted in response to exercise (including myokines, hepatokines or adipomyokines) can also generally be referred to as myokines, however they are most commonly classified due to their main origin and can be also called “exercise-induced cytokines’’ family.

The first cytokine classified as a myokine was interleukin 6 (IL-6) [1,5]. It is expressed acutely during exercise, promotes glycogen breakdown, increases glucose transporter type 4 (GLUT4) expression and glucose uptake in myocytes, enhancing insulin sensitivity [6,7]. In adipocytes, IL-6 induces lipolysis and free fatty acid oxidation [8,9]. IL-6 also facilitates muscle growth, regulating the function of satellite cells [10,11]. The first evidence of IL-6 response to exercise was published by Northoff et al. in 1991, who reported an increase in IL-6 concentration after a marathon run [12]. Further studies demonstrated that the IL-6 increase after exercise is attributable predominantly to skeletal muscle production [13,14,15,16,17,18,19]. We refer readers interested in IL-6 to the literature reviews by Pedersen et al. and Febbraio and Pedersen [20,21].

Irisin is another well studied exercise-induced cytokine, although classified mainly as a myokine or adipomyokine [22,23,24,25,26]. The muscles and adipose tissue are considered the principal source of irisin [27,28,29,30]. It has been hypothesized that irisin secreted from muscles after exercise causes so-called “browning” of the white adipose tissue. The resulting conversion of white into the beige adipose tissue, with similar thermogenic features to brown adipose tissue, raises energy expenditure [22]. Up to date, irisin has been summarized in two meta-analyses—one (Fox et al.) considering an acute bout of exercise and another (Qiu et al.) regular exercise training [23,24]. Immediately following an acute bout of exercise, irisin concentration increases substantially in adults. [23]. In contrast, randomized control trials have shown that regular training reduces the circulating irisin concentration. Evidence from non-randomized studies is inconclusive [24].

Myokines, which are perhaps the most recognizable subgroup of exercise-induced cytokines, have been recently reviewed in several aspects. Lee and Jun et al. focused on myokine activity’s molecular basis and their general functions in humans [31]. Eckel summarized the muscle-adipose, muscle-liver, muscle-bone, muscle-beta cell crosstalks, and myokines’ involvement in the anti-inflammatory effects of physical activity counteracting insulin resistance and metabolic abnormalities caused by obesity and type 2 diabetes [32]. Fiuza-Luces et al. presented an impact of physical activity on the cardiovascular system, including myokines as possible mediators of beneficial physical exercise effects in people [33]. Chen et al. focused on tissue crosstalk mechanisms, highlighting that myokines mediate the effects of muscle work on whole-body metabolism [34].

**Figure 1:**
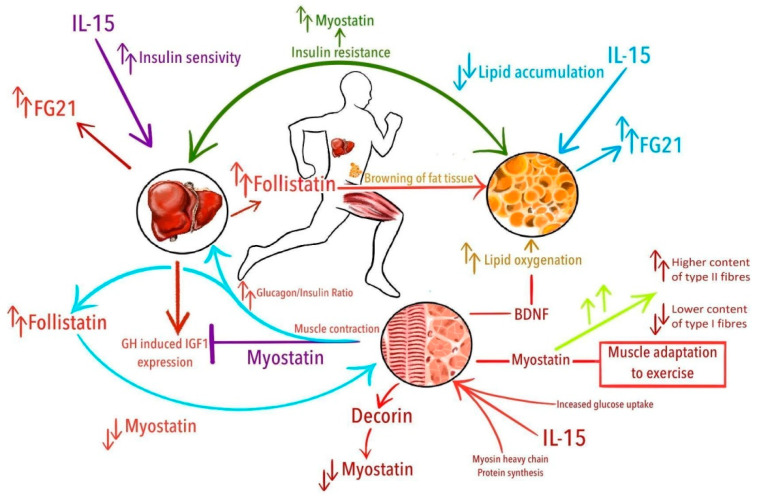
Potential relations of exercise-induced cytokines with physical activity and accompanying phenomena. From the top in clockwise manner: Overall insulin resistance is related with increased levels of myostatin; IL-15 decreases lipid accumulation; muscle-contraction induced increase of brain-derived neurotrophic factor (BDNF) drives lipid oxygenation; increased levels of myostatin are related with higher content of type II fibers and lower content of type I fibers and probably are associated with muscle adaptation to exercise; interleukin 15 (IL-15) stimulates synthesis of myosin heavy chain proteins and increase glucose uptake in the muscle; decorin binds with myostatin and downregulates its activity; in the liver, myostatin suppresses growth hormone (GH)—induced insulin-like growth factor 1 (IGF-1) expression; increased glucagon/insulin ratio after exercise leads to increased production of follistatin in the liver, which suppress myostatin activity; fibroblast growth factor 21 (FGF21) is produced mainly by the liver; IL-15 improves insulin-sensitivity; follistatin promotes browning of white adipose tissue.

New exercise-induced cytokines and exercise-related activity of already known proteins are being discovered continuously. We selected the cytokines with evidence-based relation to exercise. Myostatin (MSTN) is considered to be the most potent negative regulator of muscle mass. Conversely, follistatin (FST) and decorin (DCN) are direct antagonists of MSTN. Although the brain-derived neurotrophic factor (BDNF) concentration is strongly associated with cognitive function, it is also linked with regular physical activity. Fibroblast growth factor 21 (FGF21) and FST are produced mainly by the liver and both are considered hepatokines, nonetheless, their concentration alters in response to exercise classifying them as exercise-induced cytokines [35]. For this reason, we have included FGF21 and FST in our review as well. Finally, Interleukin 15 (IL-15) is a cytokine from the same class as myokines protoplast—IL-6; however, it is less known and deserves attention. A more detailed list of different functions and locations of expression of MSTN, FST, DCN, BDNF, FGF21, and IL-15 is given in Table 1. Figure 1 illustrates the potential relations of exercise-induced cytokines to physical activity and accompanying phenomena.

In this review, we summarize the physiological role of MSTN, FST, DCN, BDNF, FGF21, and IL-15, emphasizing the effect of various exercise and training protocols on their serum/plasma concentrations in healthy people.

## 2. Materials and Methods

Using the search engines PubMed and Google Scholar, we searched for the following phrases: “myokines and exercise”, “myokines and physical activity”, “myostatin and exercise”, “follistatin and exercise”, “decorin and exercise”, “brain-derived neurotrophic factor and exercise”, “interleukin 15 and exercise”, “fibroblast growth factor 21 and exercise”, “myostatin and physical activity”, “follistatin and physical activity”, “decorin and physical activity”, “brain-derived neurotrophic and physical activity”, “interleukin 15 and physical activity”, “fibroblast growth factor 21 and physical activity”.

The final selection was non-systematic. We reviewed papers that have been published only in peer-reviewed English-language journals over the last decade. Studies performed in healthy people were our primary focus. As the data presentation differed between the selected studies (absolute values, % of change, area under the curve, charts), the tables’ data differ in terms of units. If possible, we included the results in absolute values.

## 3. Results and Discussion

### 3.1. Myostatin

Over 20 years ago, McPherron et al. discovered myostatin (MSTN), earlier referred to as growth differentiation factor 8 (GDF-8) [66]. MSTN belongs to the transforming growth factor β (TGF-b) superfamily that controls growth and differentiation. In humans, the TGF-b superfamily comprises over 30 members that act through cell membrane receptors and further induce and integrate pathways regulating transcription in the nuclei [67]. Elliot et al. have reported that MSTN circulates in the bloodstream either in a bound (to its own propeptide or inhibiting molecules like FST) or an unbound (free) form. These forms determine biological activity of MSTN (unbound = active; bound = inactive) [68].

Schuelke et al. have identified a loss-of-function mutation in the *MSTN* in a child with muscle hypertrophy and shown for the first time that MSTN is relevant to muscle mass regulation in humans [69]. Lee et al. (2016) and Lee et al. (2010) described both paracrine and endocrine MSTN influence on muscle mass. The subtle endocrine effect of MSTN modulated whole-body metabolism [40,70]. In response to exercise, MSTN binds to the cell membrane receptors initiating the microtubule-associated protein kinase (MAPK) cascade. By causing the nuclear accumulation of SMAD (main signal transducers for receptors of the TGF-B superfamily) proteins, it activates genes involved in skeletal muscle adaptation to exercise [71,72]. Covington et al. found an association between high MSTN concentration, lower content of type I slow-twitch muscle fibers and higher content of type II fast-twitch fibers in people [73]. MSTN is involved in muscle atrophy, satellite cell activation, proliferation and differentiation, cell survival, and protein synthesis [37]. Hittel et al. have observed a positive association between MSTN concentration and insulin resistance [74]. However, MSTN’s influence on carbohydrate metabolism remains unclear and requires further investigation. Studies examining the effects of exercise on MSTN are inconclusive—Table 2.

MSTN serum/plasma concentration increases after acute exercise [75,78,79,80]. A decrease in serum MSTN was reported in one study, but this could be the effect of late blood sampling (24 h after the exercise), i.e., much later than in other studies. [77]. No exercise-induced change in MSTN was reported in a study with apparently insufficient muscle stimulus during an exercise, as participants trained only with their body mass. [76].

MSTN is also expressed in non-muscle cells and tissues. Kerschan et al. examined a group of highly trained long-distance runners after completing an ultramarathon [78]. They proposed that increased serum MSTN be a consequence of general inflammation accompanied by a release of other cytokines that might be responsible for the stimulation of MSTN synthesis. Another hypothesis would be the release of MSTN by injured muscles. Nevertheless, we have not found any reports supporting an association between MSTN concentration and muscle injury markers (e.g., myoglobin, creatine kinase, lactate dehydrogenase). MSTN concentration increases immediately after exercise and returns to baseline within 24 h of recovery.

Regular exercise effects were investigated in a few studies, ranging from five weeks to six months [68,76,81,82]. Studies reported a decrease [76,81], an increase [68] or no change [82] in MSTN basal concentrations. Contradictory results may be due to the differences in study groups, including age and sex, or the type, duration, and intensity of analyzed training.

### 3.2. Follistatin

Follistatin (FST), discovered by Robertson et al. in 1987, is a glycoprotein belonging to the TGF-b superfamily [83]. FST can suppress the follicle-stimulating hormone (FSH). In humans, FST is secreted by several organs and tissues, including gonads, skeletal muscles, liver, and adipose tissue. It circulates in three isoforms: Fst288, Fst303 and Fst301 [84,85]. According to Hansen et al., FST is mainly attributable to liver secretion and regulated by the glucagon-to-insulin ratio in response to exercise [43]. Even though FST liver secretion is induced by muscle contraction and, therefore, is considered a hepatokine, some authors also list it as a myokine [80,86]. Regardless of the label, FST belongs to the exercise-induced cytokines family.

FST is an antagonist of MSTN and other TGF-b superfamily members and prevents these proteins from binding to the activin IIb receptor [47,87]. FST regulates muscle cell metabolism, differentiation, and growth indirectly through inhibition of MSTN effects or, as reported by Winbanks et al., directly by mechanisms independent from MSTN [44,45]. In humans under increased energy demand, for example, after acute exercise or prolonged fasting, FST is probably stimulated by an increased glucagon-to-insulin ratio [86,88]. Studies examining the effect of physical exercise on FST are consistent, demonstrating its increase after exercise—Table 3.

The studies investigating relations between exercise and FST focus primarily on acute rather than long-term effects. All types of exercise, including resistance [79,81,82,89,90], endurance [43,78,80,89,91,92] and HIIT [68,79,89] raised plasma/serum FST after an acute bout, despite the variety of used protocols. An increase in FST concentration ranges from around 5% to 500%. The strongest response has been found in younger subjects performing the exercise with higher intensity. These features may be the factors limiting an increase in FST serum/plasma concentration in other studies. FST concentration peaks at 3–4 h post-exercise, and then it decreases; however, in some studies, an elevated concentration was observed for 72 h.

Effects of regular exercise were investigated in studies based on training lasting from 2 weeks to 6 months. In the studies considering serum/plasma FST, regular physical activity increases its basal concentration, regardless of the training duration and the type of performed activity. However, it must be highlighted that the participants in these studies were predominantly middle-aged and elderly.

### 3.3. Decorin

DCN is a small, leucine-rich proteoglycan encoded by the *DCN* gene involved in the collagen-fibril assembly. It binds to multiple cell surface receptors, modulates tumor suppression, stimulates autophagy and inflammation, and inhibits angiogenesis and tumorigenesis [50]. DCN is secreted by skeletal muscle cells. As a myokine, it promotes skeletal muscle hypertrophy by binding with MSTN and regulates muscle growth upon physical exercise [93]. A summary of studies on DCN is presented in Table 4.

Few studies have explored the potential role of DCN in physical exercise. Studies presented in Table 4 were performed in small groups of subjects (<15 individuals), except for the investigation by Micielska et al. [76]. Kanzleiter et al. reported that plasma DCN concentration increased in humans performing acute resistance exercises. They also observed an enhanced secretion of DCN from in vitro contracting human myotubes in response to chronic endurance training. [93]. Knuiman et al. studied 13 young and healthy adults who underwent endurance and resistance exercise sessions [94]. DCN concentration did not differ significantly between the baseline and after the training. However, although not significant, an increasing trend in the plasma DCN concentration was found at 1, 2, and 3 h after exercise, compared with the baseline values. Knuiman et al. drew blood from subjects one hour after exercise, whereas the peak release of protein is right after the exercise [93]. Bugera et al. found that plasma concentration of DCN immediately post-exercise was higher than immediately pre-, 1 h post-exercise, and 24 h post-exercise. [95] On the other hand, Micielska et al. reported that high-intensity circuit training for five weeks did not change the resting plasma concentration of DCN in women [76]. This discrepancy might be explained by differences in applied exercise protocol and timing for drawing blood for DCN measurement [76].

In conclusion, the effects of exercise on DCN are not well explored, and their understanding requires future research on larger groups of people. It is, however, known that DCN binds extracellularly to MSTN. Consequently, MSTN’s regulatory effects are inhibited, leading to muscle fiber hypertrophy [93,96].

### 3.4. BDNF

Brain-derived neurotrophic factor (BDNF) is a protein encoded by the *BDNF* gene. BDNF, first isolated in 1898 by Yves-Alain Barde and Hans Thoenen [97], is part of a larger family of neurotrophins primarily responsible for developing neurons [51]. BDNF is essential in the autocrine mediation of adult dorsal root ganglion neuron survival [98]. This neurotrophin is involved in numerous neurophysiological processes, including the control of neuronal and glial cell development and the activity-dependent regulation of the synaptic structure and its maintenance, i.e., processes critical for memory and cognition [52].

The origin of BDNF in human serum is still not fully understood. Recent studies have found that megakaryocytes are the primary source of BDNF in platelets and serum [99], suggesting that serum BDNF concentration does not reflect its brain level. The variations in BDNF concentration during physical exercise may reflect different degrees of platelet activation [100]. On the other hand, Rasmussen et al. reported that the brain contributed 70–80% of circulating BDNF both at rest and during exercise [101]. Non-neurogenic tissues, including skeletal muscle, also release this protein. Exercise increases BDNF in the brain, plasma, and skeletal muscles. However, muscle-derived BDNF appears not to be released into the circulation [53]. BDNF is a contraction-inducible protein enhancing lipid oxidation in skeletal muscles via AMP-activated kinase activation [53]. The relation of BDNF to exercise is shown in Table 5.

Most of the analyzed studies report an increase in serum BDNF after bouts of exercise, including aerobic, anaerobic, or more prolonged efforts. In some studies, however, BDNF concentration remained unchanged or decreased after exercise. Studies analyzed BDNF concentration changes after an acute bout of exercise and a more extended training protocol, whereas some only one of these factors. Most studies measured serum BDNF concentration, and one study measured plasma BDNF concentration. [108]. Serum BDNF measurement is favoured over the EDTA-plasma for its higher reliability [114].

Szuhany et al. performed a meta-analysis [102]. Fourteen studies examined BDNF concentration changes after a single session of exercise, eight studies immediately post-exercise in a design evaluating the effects of a regular training program, and 13 studies at rest but during a longer regular training. Every bout of exercise causes an increase in BDNF concentration, and the magnitude of this effect can be enhanced by prolonged training. Resting BDNF has been reported to rise after an exercise program. Szuhany et al. found that BDNF after exercise increased to a lesser extent in women than in men. Hakansson et al. found that BDNF increased after exercise in older adults aged 65–85 years [103]. Tsai et al. highlighted that serum BDNF secretion after a bout of exercise depended on the exercise intensity, as more forceful effort caused a higher BDNF increase [107]. On the other hand, Church et al. reported that BDNF increased after an acute bout of resistance exercise in a group of experienced weight lifters regardless of the training protocol (high-intensity, low-volume training, or high-volume moderate-intensity training). No significant differences in BDNF concentration were found between the training protocols [108]. Moreover, BDNF concentration increased after a 7-week training [108]. Roh et al. [113] observed that serum BDNF increased after a 16-week taekwondo training program.

Murawska et al. reported that resting BDNF increased after the 12-week CrossFit program in both men and women. However, before the 12-week CrossFit program, BDNF increased directly after the Wingate and progressive tests, while after the training was completed, BDNF decreased right after the Wingate and progressive tests [106]. Different physiological adaptation levels to exercise before and after the training may play a vital part in BDNF concentration changes after a single exercise.

Marquez et al. reported that BDNF concentration increased similarly during high-intensity interval training and a continuous exercise protocol. However, BDNF increase during exercise was higher in the high-intensity protocol. In this study, BDNF concentration was measured eight times during exercise, giving a unique insight into this protein’s exercise-related kinetics. BDNF gradually increased during the 20 min exercise, with maximal concentrations found at the end of the activity. It quickly decreased to basal values after 20 min of rest [109]. Fortunato et al. [105] confirmed that BDNF peak after exercise is relatively short, and BDNF returned to normal values within 2 h of recovery.

Antunes et al. [111] observed that exercise raised BDNF independently of the intensity protocol; however, the largest BDNF increase was noted after the high-intensity training of >90% VO2max. Additionally, they reported that BDNF increase was higher in subjects with lower than higher physical fitness levels during the short-time high-intensity exercise. This finding suggests that post-exercise growth in BDNF depends on the physical fitness level. This finding can be a possible explanation for some of the previous results of some reduction in BDNF after exercise (Wagner et al. [110], Murawska et al. [106]). In contrast to most reports showing a post-exercise/training increase in BDNF, Wagner et al. found a significant exercise-induced decline in BDNF both before and after a 6-week aerobic physical training [110]. No response of baseline BDNF to longer physical exercise protocols has been noted by Antunes et al. [111] and Wagner et al. [110].

Most of the studies were performed in small groups of less than 20 participants [103,105,106,109,113], usually with no or just a few women [105,107,108,109,110,111,112,113]. Various types of exercise with BDNF have been analyzed so far. Subjects exercised on a cycle ergometer [104,109,111], treadmill [112], underwent strength exercises [105], high intensity interval training [106,108,109] or other types of exercises like general workout or taekwondo [102,103,107,110,113]. The exercise duration was also diverse ranging from 20 to 35 min for acute exercise to 6 to 16 weeks for the regular training programs. Different exercise loads during endurance exercise were applied from 40% to 95% VO2max. These differences make it difficult to compare the observed effects of the studies. On the other hand, different exercise types and their duration evoked similar effects—an increase in BDNF concentration after exercise, with few exceptions in rare situations [106,110,111].

In conclusion, BDNF rises after physical exercise in healthy adults after acute bouts of exercise and after regular exercise [103,104,105,106,107,108,109,112,113]. A more significant increase in BDNF concentration is demonstrated after high-intensity training [104,107,109] and more extended training lasting at least 12 weeks [106,112,113]. However, a single bout of strength exercise also increases BDNF [103,104,105,106,107,108,111]. Fitness level influences BDNF concentration changes, but results are inconsistent. Most recently, authors report that subjects with lower physical fitness levels had a more significant increment in BDNF than higher fitness level subjects [106,110,111].

### 3.5. Fibroblast Growth Factor 21 (FGF21)

Fibroblast growth factors (FGFs) comprise a group of cell-signaling proteins with mitogenic activity. They facilitate cell differentiation, migration, and survival [54]. Fibroblast growth factor 21 (FGF21) belongs to the subfamily of so-called endocrine FGFs secreted into the bloodstream and expressing metabolic and endocrine functions [115]. The liver secretes most of FGF21 in response to exercise. However, FGF21 is also expressed in adipose tissue, skeletal muscle, and pancreas [116,117,118]. Similar to FST, FGF21 is an exercise-induced cytokine which primarily originates from the liver. Therefore, FGF21 is considered as a hepatokine, though due to its exercise-dependent character and activity, it can also be referred to as either adipokine or myokine [119]. It acts through the FGFR receptor in the presence of the cofactor beta-klotho—its tissue expression determines the target organs for the FGF21 [120]. FGF21 modulates metabolic adaptation to stress [117,121,122,123], decreases serum glucose and lipid concentrations, and improves insulin sensitivity [124,125]. Data on the relation between FGF21 and exercise are summarized in Table 6.

In general, serum FGF21 concentration increases in response to acute bouts of endurance exercise [79,91,92,126,127,128,129]. However, Cuevas-Ramos et al. and Taniguchi et al. observed no significant changes in serum FGF21 after a single session of endurance exercise [130,131]. These discrepancies may result from lower training intensity and shorter duration compared to other studies. According to Kim et al. and Wills et al., FGF21 rises proportionally to the training intensity [92,126]. In the study by Cuevas-Ramos et al., the average training duration was approximately 14 min, whereas, in Taniguchi et al., the exercise lasted 30 min at 70% VO2max [130,131]. Other factors potentially influencing the obtained results are the participants’ age and gender. Most studies in which IGF21 increased were carried out in young men. By contrast, Cuevas-Ramos et al. investigated women and Taniguchi et al. older men. 

Moreover, FGF21 serum concentration rises gradually after acute endurance exercise with no or slight alterations observed immediately after exercise and peak values measured one-hour post-exercise [79,91,92,126,127,128,129]. Few studies assessed further time points, though FGF21 concentration tends to normalize quickly in the resting period [128], reaching even lower than baseline values 24 h post-exercise [131]. In a recent meta-analysis, circulating FGF21 increased immediately after exercise, reached maximal concentration at one hour of recovery and returned to baseline within three hours of recovery [132].

Janssen Duijghuijsen et al. studied well-trained individuals who cycled on two occasions separated by one week [129]. The first exercise increased serum FGF21, whereas the retest performed after a week did not affect FGF21 concentration. The authors suggested this may reflect an adaptation to exercise-induced cellular stress that stimulated FGF21 during the first trial.

The data on the influence of regular endurance training on serum FGF21 are scarce. Cuevas-Ramos et al. observed a significant increase in FGF21 concentration after two weeks of regular physical activity. However, their study lacks information on the exact time point of blood sampling after a complete intervention [130]. It is unclear whether blood samples were taken before or after the last training session, and consequently if acute exercise might have influenced the results. By contrast, Taniguchi et al. reported a significant decrease in serum FGF21 in older men after five weeks of regular cycling [133]. The authors hypothesize that prolonged exercise reduced the so-called FGF21 resistance, similarly to insulin resistance, and consequently decreased FGF21 serum concentration. The presented studies on the effects of regular physical activity on FGF21 differed significantly in the training duration, intensity, and participants’ age and sex, which might influence the obtained results.

Three of the reviewed studies focused on resistance training [79,128,134]. Results indicate that serum FGF21 remains unchanged shortly after acute resistance exercise, regardless of its intensity and involved muscle mass [128,134]. However, it may increase in the late phase of recovery [79].

### 3.6. Interleukin 15

Interleukin 15 (IL-15) was discovered in 1994 as a T-cell growth factor [58,135]. Its expression was identified in various cells and tissues, including cells of monocyte/macrophage lineage and other antigen-presenting cells, skeletal and heart muscle, placenta, kidney, lung, bone marrow, and thymus [59,135]. IL-15 regulates cell proliferation, maturation, and the extent of the cellular response in the immune system. It also exerts pleiotropic functions in lipid and glucose metabolism [62,63]. IL-15 administration prevents fatty liver development induced by a high-fat-fed diet and reduces serum glucose and insulin concentrations leading to improved insulin sensitivity [64,65]. In myocytes, IL-15 enhances basal and insulin-induced glucose uptake and GLUT4 translocation to the cell membrane [136,137]. IL-15 is engaged in transducing the beneficial effects of exercise on health. IL-15 promotes muscle anabolism through stimulation of myosin heavy-chain protein synthesis in mature muscle fibers [61]. Although earlier studies found that exercise interventions did not elicit IL-15 serum concentration changes, [138,139,140] recently demonstrated that an acute bout of resistance exercise and regular endurance training affect IL-15 [141,142,143]. Summary of studies on IL-15 and exercise or training are presented in Table 7.

Acute resistance exercise stimulates IL-15 production [94,144,145]. Its increase occurred within the first hour of recovery and was not affected by pre-exercise availability of carbohydrates or fat. However, Perez-Lopez et al. noted that an increase in serum IL-15 was not associated with an increased muscle protein synthesis, questioning the role of this myokine in exercise-induced muscle hypertrophy [145].

In contrast, Bugera et al. and Fortunato et al. reported no influence of a single session of resistance exercise of legs on serum IL-15 [95,105]. They speculated that exercise limited to lower extremities might be insufficient to induce myokine secretion. However, other studies demonstrated that IL-15 raised in response to similar resistance exercise [94,145]. He et al. investigated whole-body resistance training and reported no changes in IL-15 concentration. Only one study focused on the long-term effects of regular resistance training and demonstrated an increase in IL-15 concentration after completing a 12-week whole-body resistance training protocol [146].

The mentioned studies differed in subjects’ fitness level, which possibly affected the IL-15 response to exercise. Bazghir et al. and Kapilevich et al. acknowledged that fitness level might modulate IL-15 concentration [147,148]. Higher baseline IL-15 was observed in both resistance and endurance-trained athletes compared to untrained individuals [148]. A higher increment of serum IL-15 upon exercise was observed in athletes after the eccentric than concentric phase emphasized resistance exercises. The IL-15 increment found in athletes doing resistance exercises was also higher than in untrained subjects [147]. On the other hand, Kapilevich et al. reported that endurance training did not influence IL-15 concentration in either trained or untrained participants.

Perez-Lopez et al., Yargic et al. and Micielska et al. studied cycling sprint and long-distance trail running and found upregulated IL-15 secretion [76,149,150]. Micielska et al. demonstrated an altered response of IL-15 to high-intensity circuit training following 5-week regular training [76]. After the first training session, serum IL-15 declined below its baseline values. After the last training, IL-15 increased. These results support the hypothesis that IL-15 increases along with an improving fitness level. An acute bout of high-intensity interval endurance training did not change IL-15 concentration in sedentary subjects [79].

Nishida et al. demonstrated no change in IL-15 concentration in older women after 12 weeks of regular aerobic activity, namely, bench step exercise [151]. However, the training was not supervised, which might have influenced the results. Similarly, Roh et al. reported no change in IL-15 concentration in overweight and obese adolescents undergoing a 16-week taekwondo training [113]. Participants’ body composition might have affected this study results as IL-15 concentration correlates with fat mass, and its response to exercise in overweight and obese individuals may be altered [152].

## 4. Conclusions

Research on exercise-induced cytokines has many limitations and thus is not easy. The most common limitation is a small number of studied individuals who usually have distinct baseline characteristics like sex (mainly men), age (usually younger participants), or race. Another is the use of various protocols for physical activity—it complicates the comparison of obtained results between different studies. The next limitation is related to the technical specifications of cytokine measurement. Some are measured in either blood plasma or serum, whereas others in muscle biopsies. Regardless of the mentioned problems, a certain generalization of the exercise effects on cytokines is possible.

There is no doubt that exercise affects many organs and tissues, which release various cytokines in response. Some are directly secreted by contracting muscles (MSTN, DCN, BDNF, IL-15) while others (FST, FGF21) by non-myocyte cells, for example, hepatocytes or adipocytes. These exercise-induced cytokines regulate muscle differentiation, growth, and remodeling in response to the whole body’s exercise and metabolism.

The change in the concentration is cytokine-specific and also depends on various features of the exercise. An important feature is a type of exercise like endurance, resistance, dynamic power, or HIIT. Another is related to the metabolism mode, such as aerobic, anaerobic, or mixed. An additional feature is the exercise’s intensity. It can be measured as the percentage of VO2max for the endurance activity, the percentage of the one-repetition maximum for the resistance exercise, or the percentage for the estimated maximal heart rate for any form of physical activity. The amount and duration of exercise impact the release of the cytokines as well. Short-lasting or a single bout of an acute exercise has a different effect on cytokines than prolonged activity or repeated regular training. The baseline fitness level may modify the type, and extent of cytokine response to physical activity as some of these proteins are secreted or not, depending on whether someone is a worse or better fit.

In this review, we have focused mainly on the physiology of exercise-induced cytokines. Studying these proteins and their relation to exercise, although challenging, is very interesting. It helps to understand many aspects of exercise physiology and should be useful in the research on muscle pathology or limited exercise tolerance. As some of the cytokines are involved in muscle growth and differentiation of progenitor cells into myocytes, studying them is promising in regenerative medicine. Investigation of other exercise-related cytokines regulating energy, lipid, carbohydrate, or protein metabolism might be valuable in researching several metabolic diseases and conditions like diabetes, obesity, malnutrition, or cachexia. Still, many gaps in the knowledge about exercise-induced cytokines (i.e., myokines, hepatokines, adipomyokines) exist. New studies and findings gradually fill these gaps.

## Figures and Tables

**Table 1:** Potential functions of selected cytokines and location of expression.

Cytokine	Potential Function	Location of Expression [36]
**Myostatin**	Muscle protein synthesis and degradation [37] Downregulation of muscle structural genes and myogenic factors [37] Inhibition of muscle cell proliferation and differentiation [37] Inhibition of activation of the AKT/mTOR pathway [37] Regulation of the number of muscle fibers in embryogenesis [37] Inhibition of osteoblastic differentiation [38] Alternating glucose metabolism [39,40] Potential regulation of the GH/IGF-1 axis [41] Promoting of ROS production [42]	Muscle tissues Adipose and soft tissue Brain Eye Endocrine tissues Gastrointestinal tract Skin Bone marrow Blood
**Follistatin**	Suppression of follicle-stimulating hormone [43] Inhibition of myostatin [44] Myostatin independent muscle growth regulation [45] Browning of white adipose tissue [46] TGF-beta family signaling inhibition [47] Inhibition of extracellular matrix turnover [48] Insulin-dependent regulation of glucose uptake [49] Reduction of ROS production [47]	Liver and gallbladder Female tissues Pancreas Muscle tissues Adipose and soft tissue Skin Bone marrow Blood
**Decorin**	Promotion of muscle hypertrophy by binding with myostatin [50] Stimulation of autophagy and inflammation [50] Inhibition of angiogenesis and tumorigenesis [50] Modulation of tumor suppression [50]	Female tissues (especially ovary, placenta, breast, endometrium) Muscle tissues Adipose and soft tissue Lung
**Brain-derived neurotrophic factor**	Development and survival of adult dorsal root ganglion neurons (motor neurons) [51] Development of neuronal and glial cells [52] Regulation of the synaptic structure and its maintenance—critical for memory and cognition [51,52] Exercise-induced neuroplasticity—may contribute to improvement in locomotor function [51] Remodeling of injured axons [52] BDNF produced in skeletal muscle enhances fat oxidation [53]. A vital component of the hypothalamic pathway that controls body mass and energy homeostasis [53]	The central and peripheral nervous system (hippocampus, cortex, and basal forebrain) Skeletal muscles Retina Kidney Saliva Prostate Megakaryocytes Others
**Fibroblast growth factor 21**	Cell differentiation, migration and survival [54] Lipid metabolism regulation [55] Glucose metabolism regulation [55] Adaptive response to starvation [56]. Browning of white adipose tissue [57]	Liver Adipose tissue Skeletal muscle Pancreas
**Interleukin 15**	Immune cell proliferation, differentiation and maturation [58,59] Immune response regulation [60] Muscle protein synthesis [61] Adipose tissue reduction [62,63]. Glucose metabolism regulation [64,65]	Skeletal and heart muscle Placenta Monocytes/macrophages Bone marrow Thymus Lung Liver Kidney

Abbreviations: ROS—reactive oxygen species; IGF-1—insulin-like growth factor 1; GH—growth hormone; TGF—transforming growth factor; AKT/mTOR—protein kinase B/mammalian target of rapamycin signaling pathway.

**Table 2:** Effects of exercise on myostatin.

Forms of Exercise	Group Characteristics	Blood Samples	Effect of Exercise on the Cytokine Concentration	References
**Acute exercise effects**				
HIIT. Four 30-s Wingate anaerobic tests (WAnTs) with a 4-min rest on cycle ergometer between consecutive exercise bouts	Men aged 18–24 years, 10 Kick-boxers and 10 sedentary-lifestyle (*n* = 20)	Blood samples—before, just after, 3 and 6 h post-test	Increase of MSTN serum concentration, kick-boxers and sedentary, respectively: PRE = 2652.3 ± 960.9 pg/mL and 2501 ± 78.3 pg/mL After = 2914.5 ± 1011.6 pg/mL and 2939.1 ± 839.2 pg/mL 3 h post = 2608.9 ± 890.6 pg/mL and 2849.5 ± 1028.6 pg/mL 6 h post = 2698.2 ± 1004.2 pg/mL and 2717 ± 764 pg/mL	[75]
HICT with body mass, 3× per week for 5 weeks. One HICT session consisted of 3 circuits with 2-min breaks between them. Each HICT training consisted 9 exercises with one’s body as a workload performed as follow: jumping jacks, pushups, sit-ups, side plank, squats, plank, running in place, lunges, and push-ups with rotation	Women, aged 40 ± 11 years, inactive (*n* = 20). Age subdivision: young < 30 years (*n* = 11) and middle-age > 30 years (*n* = 9)	Blood samples—before and 1 and 24 h after the first and last session	No change	[76]
A single bout of resistance exercise. RE session targeted the upper and lower body, with three sets of 15 repetitions on seven machines (chest press, pull up, leg press, shoulder press, knee extension, knee flexion, and elbow flexion), each lasting 30 s. All exercises were performed at 55 % of the subjects’ 1 RM with 2-min rest intervals between sets	Men, aged 22.1 ± 2.1 years, experienced in weight training (*n* = 12)	Blood samples—before and 24 h after the exercise.	Decrease of MSTN serum concentration (Baseline = 9.12 ± 0.36 ng/mL; Post-exercise = 8.8 ± 0.36 ng/mL)	[77]
Ultramarathon—foot race of 246 km distance from Athens to Sparta; time limit <36 h	18 men and 1 woman, aged 41–48 years, Highly trained long-distance runners (*n* = 19)	Blood samples—the day before a run, just after the race, and 3 days after	Increase in MSTN serum concentration (Before = 23.73 ng/mL (21.16–28.28) and just after = 26.73 ng/mL (21.22–31.68))	[78]
HIIT1: two sets of six 30 s treadmill running bouts at Vmax eliciting the VO2max, with 90 s of active recovery (50% of Vmax) between bouts and 4 min of passive recovery between sets. HIIT2: five 4 min bouts at 90% Vmax, with 4 min of active recovery (50% of Vmax) between bouts	Men, aged 23 ± 2 years, sedentary (*n* = 17)	All blood variables were analyzed before (baseline) and immediately after each training session and 1, 3, 24, 48, and 72 h after each session.	Increase in MSTN serum concentration. HIIT1 to ~130% of the basal value. HIIT2 to ~135% of the basal value.	[79]
Resistance Training. Seven types of exercises targeting all the main muscle groups. Four sets of 8–10 repetitions at 70–75% of 1 RM with 60–90 s of rest between exercises and for 4 min between sets.	Men, aged 23 ± 2 years, sedentary (*n* = 17)	All blood variables were analyzed before (baseline) and immediately after each training session and 1, 3, 24, 48, and 72 h after each session.	Increase in MSTN serum concentration to ~145% of the basal value.	[79]
Session 1: 45 min of treadmill running at the intensity of the anaerobic threshold (AT; 85 ± 8% of VO2max) Session 2: 45 min of treadmill running at the intensity of the maximal fat oxidation (FATmax; 52 ± 14% of VO2max)	Men, aged 23 ± 1 years, sedentary (*n* = 14)	Blood variables were analyzed at baseline, immediately upon session termination (post 0 h), and 1, 3, 24, 48, and 72 h after each session, respectively.	Increase in MSTN serum concentration after AT session (Peak% = 136 ± 18). No change in FATmax.	[80]
**Regular (chronic) exercise effects**				
Supervised training three times a week, separated by at least 48 h for 8 weeks. training included three sets (50–80% 1 RM) per exercise in weeks 1–4 and four sets per exercise in weeks 5–8.	Men, aged 40–53 years, sedentary Upper-body (*n* = 10) Lower-body (*n* = 10) Upper + Lower body (*n* = 10)	Blood samples—48 h before the first session and 48 h after the last session	Decrease of MSTN serum concentration in LB and UP + LB LB = (−0.11 ng/mL (95% CI −0.16 to −0.06)) UB + LB = (−0.36 ng/mL (95% CI −0.48 to −0.19))	[81]
HIIT. One session every 5 days for 6 weeks—nine sessions in total. Each session consisted of 6 × 30 s sprints at 40% predefined peak power output interspersed with 3 min active recovery on a cycle ergometer	Older Men. LEX = 11 active exercisers aged 62 ± 6 years, masters in sports; SED = 13 sedentary lifestyle aged 64 ± 6 years (*n* = 24)	Blood samples—before and after completing the program.	Trivial increase of total MSTN serum concentration in SED (PRE = 4217 ± 317 pg/mL; POST = 4163 ± 337 pg/mL). Moderate increase of total MSTN serum concentration in LEX (PRE = 3394 ± 391 pg/mL; POST = 3678 ± 438 pg/mL). Trivial increase of free MSTN in SED and LEX (PRE = 1182 ± 372 pg/mL and 1159 ± 418 pg/mL respectively; POST = 1203 ± 533 pg/mL and 1224 ± 404 pg/mL respectively)	[68]
HICT with body mass, 3× per week for 5 weeks. One HICT session consisted of 3 circuits with 2-min breaks between them. Each HICT training consisted 9 exercises with one’s body as a workload performed as follow: jumping jacks, pushups, sit-ups, side plank, squats, plank, running in place, lunges, and push-ups with rotation	Women, aged 40 ± 11 years, inactive (*n* = 20). Age subdivision: young < 30 years (*n* = 11) and middle-age > 30 years (*n* = 9)	Blood samples—before and 1 and 24 h after the first and last session	Decrease of MSTN serum concentration (more pronounced in middle-aged)	[76]
Supervised, resistance band training designed to train all major muscle groups based on ACSM guidelines—twice a week for 6 months	Untrained Women > 65 years. Resistance training (*n* = 33); Resistance training and supplementation (*n* = 28)	Blood samples—baseline, after 3 months and 6 months of intervention	No changes	[82]

Abbreviations: 1 RM—one repetition maximum, the maximum amount of weight that a person can lift for one repetition; HIIT—high-intensity interval training; VO2max—the maximum rate of oxygen consumption; ACSM—The American College of Sports Medicine; HICT—high-intensity circuit training; RE—resistance exercise; Vmax—maximal speed; UB—upper body; LB—lower body; AT—anaerobic threshold; y—years old.

**Table 3:** Effects of exercise on follistatin.

Forms of Exercise	Group Characteristics	Blood Samples	Effect of Exercise on the Cytokine Concentration	References
**Acute exercise effects**				
HIIT1: two sets of six 30 s treadmill running bouts at Vmax eliciting the VO2max, with 90 s of active recovery (50% of Vmax) between bouts and 4 min of passive recovery between sets. HIIT2: five 4 min bouts at 90% Vmax, with 4 min of active recovery (50% of Vmax) between bouts	Men, aged 23 ± 2 years, sedentary (*n* = 17)	All blood variables were analyzed before (baseline) and immediately after each training session and 1, 3, 24, 48, and 72 h after each session.	Increase in serum FST in both sessions. HIIT1 ~200% of basal concentration and HIIT2 ~270% of the basal concentration in 3 h post-exercise	[79]
Resistance training: seven types of exercises targeting all the main muscle groups. Four sets of 8–10 repetitions at 70–75% of 1 RM with 60–90 s of rest between exercises and 4 min between sets.	Men, aged 23 ± 2 years, sedentary (*n* = 17)	All blood variables were analyzed before (baseline) and immediately after each training session and 1, 3, 24, 48, and 72 h after each session.	Increase in serum FST ~200% of the basal concentration in 3 h post-exercise	[79]
Session 1: 45 min of treadmill running at the intensity of the anaerobic threshold (AT; 85 ± 8% of VO2max) Session 2: 45 min of treadmill running at the intensity of the maximal fat oxidation (FATmax; 52 ± 14% of VO2max)	Men, aged 23 ± 1 years, sedentary (*n* = 14)	Blood variables were analyzed at baseline, immediately upon session termination (post 0 h), and 1, 3, 24, 48, and 72 h after each session, respectively.	Increase in serum FST. AT peak% = 359 ± 177 and FATmax peak% = 241 ± 86	[80]
45 min of walking/ running at 70% VO2max, followed by 90% of VO2max until exhaustion	80 Men divided into subgroups. Young, aged 18–35 years (*n* = 20) and Old aged 61–79 years (*n* = 20); Active—VO2 max >35 mL/kg/min (*n* = 20) and inactive—VO2 max <35 mL/kg/min (*n* = 20)	Blood was obtained 1 h before the end of the exercise, immediately after, and 1 h after completing each session.	Increase in serum FST ranging 12–21% in the general population. Young active peak% = 22.8 ± 34.7 Young sedentary peak% = 14 ± 27.8 Old active peak% = 12 ± 18.9 Old sedentary peak% = 12.4 ± 17	[89]
1: High-intensity interval exercise (HIIE), consisting of 5 × 4 min walking on a treadmill at 3 km/h alternating with 4 × 4 min running at 90% of the maximum heart rate for a total of 36 min. 2: Continuous moderate-intensity exercise (MIE), defined as a 6-min walk/run on a treadmill at 65% of maximum heart rate. 3: Resistance exercise (RE), consisting of 3 sets of 8 to 12 repetitions at 75% to 80% of 1 RM	Men aged 30–56 years, sedentary (*n* = 14)	Blood was obtained 1 h before the end of the exercise, immediately after, and 1 h after completing each session.	Increase in serum FST ranging 5–10% in HIIE and RE group	[89]
Eccentric exercise bout with the knee extensors of each leg on an isokinetic dynamometer. 2 sets of 25 maximal voluntary eccentric (lengthening) muscle actions in isokinetic mode, while a 5 min break was allowed between the sets.	Men aged 25.7 ± 1.7 years; inactive for last 6 months (*n* = 9)	Blood samples were collected before and at 6, 48, and 120 h after the eccentric exercise,	Increase in serum FST PRE = 2080.2 pg/mL (± 200.3); 6 h = 2827.2 pg/mL (± 472.6); 48 h = 2924.4 pg/mL (± 330.2); 120 h = 2144.4 pg/mL (± 177.9)	[90]
60 min of treadmill running at 60% VO2max	Men, aged 36 ± 15 years; inactive or moderately active (*n* = 11)	Trials were then initiated with a venous blood sample taken at ~09:00 (0 h), and additional samples were collected at 1, 1.5, 2.75, 4, and 7 h.	Increase in FST serum concentration with a peak at 2.75 h (~1000 pg/mL at the baseline to ~1400 pg/mL at peak)	[91]
MOD: Treadmill run at medium intensity 55% VO2max to energy expenditure 600 kcal HIGH: Treadmill run at high intensity 75% VO2max to energy expenditure 600 kcal	Men, aged 26 ± 2 years (*n* = 10)	Blood samples—before exercise, just after and 1, 2, 4, 7 h after	Increase in FST plasma concentration. AUC concentrations: PRE = 4518 ± 1148 pg/mL and 4566 ± 962 pg/mL in MOD and HIGH, respectively. POST = 8504 ± 2118 pg/mL and 9275 ± 1406 pg/mL in MOD and HIGH, respectively.	[92]
2 h of bicycle exercise at 60% of VO2max followed by 4 h of resting recovery	Men, aged 22.9 ± 0.8 years (*n* = 10)	Blood samples for analysis of hormones were obtained before a test and then every hour (one extra sample 30 min post-exercise) simultaneously from the hepatic vein and the brachial artery	5-fold increase in plasma FST in the hepatic vein and brachial artery during the first 2 h of recovery	[43]
Ultramarathon—foot race of 246 km distance from Athens to Sparta; time limit <36 h	18 men and 1 woman, aged 41–48 years, highly trained long-distance runners (*n* = 19)	Blood samples—the day before a run, just after the race and 3 days after	Increase in serum FST PRE = 300.8 pg/mL [236.4; 831.5] POST = 1211 pg/mL [849.1; 2174]	[78]
**Regular (chronic) exercise effects**				
HIIT. One session every 5 days for 6 weeks—nine sessions in total. Each session consisted of 6x30 sec sprints at 40% predefined peak power output interspersed with 3 min active recovery on a cycle ergometer	Older Men. LEX = 11 active exercisers aged 62 ± 6 years, masters in sports; SED = 13 sedentary lifestyle aged 64 ± 6 years (*n* = 24)	Blood samples—before and after completing the program.	Increase in serum FST in SED while no change in LEX. PRE = 2508 ± 628 pg/mL and 2102 ± 598 pg/mL in SED and LEX, respectively. POST = 3043 ± 676 pg/mL and 2126 ± 809 pg/mL in SED and LEX, respectively.	[68]
Supervised resistance band training designed to train all major muscle groups based on ACSM guidelines—twice a week for 6 months	Untrained women >65 years old. RT = resistance training (*n* = 33); RTS = resistance training and supplementation (*n* = 28)	Blood samples—baseline, after 3 months and 6 months of intervention	Increase in basal serum FST in the RT group PRE: 1.92 ng/mL (1.38–2.86); POST: 2.23 ng/mL (1.34–3.61)	[82]
Supervised training three times a week, separated by at least 48 h for 8 weeks. training included three sets (50–80% 1 RM) per exercise in weeks 1–4 and four sets per exercise in weeks 5–8.	Men, aged 40–53 years, sedentary Upper-body (*n* = 10) Lower-body (*n* = 10) Upper + Lower body (*n* = 10)	Blood samples—48 h before the first session and 48 h after the last session	Increase in serum FST [UB = 0.22 ng/mL (95% CI 0.16–0.38); LB = 0.24 ng/mL (95% CI 0.20–0.28) and UB + LB = 0.55 ng/mL (95% CI 0.39–0.61)]	[81]

Abbreviations: 1 RM—one repetition maximum, the maximum amount of weight that a person can lift for one repetition; HIIT—high-intensity interval training; VO2max—the maximum rate of oxygen consumption; ACSM—The American College of Sports Medicine; Vmax—maximal speed; MOD—moderate intensity; HIGH—high intensity; AT—anaerobic threshold; UB—upper body; LB—lower body; AUC—area under the curve; y—years old.

**Table 4:** Effects of exercise on decorin.

Forms of Exercise	Group Characteristics	Blood Samples	Effect of Exercise on the Cytokine Concentration	References
**Acute exercise effects**				
Strength training session of seven exercises performed in three sets at a load corresponding to 8-RM (the weight that could be lifted maximal 8 times). The exercises were leg press, leg curls, bench press, pull-down, sitting shoulder press, cable-flies, and low rowing.	Men, young, well-trained and lean (*n* = 10) (acute setting)	Before the start of the exercise, after the third set of leg press, immediately after the exercise session (0 min) as well as 30 min, 60 min, 90 min, and 120 min post-exercise	1. Plasma decorin levels were significantly increased at the end of the exercise session (~30% increase) 2. Volunteers who could push more weight displayed a higher increase of decorin levels from baseline to the end of the training session	[93]
An endurance exercise session (90 min on a cycle ergometer at 70% VO2max) in the morning and a resistance exercise session (5 × 8 80% 1 RM—repetition max) repetitions of bilateral leg press and the leg extension with two minutes of rest in between the sets) in the afternoon with a resting period of 4 h between sessions	Men, mean age 21.2 years, recreationally active (*n* = 13)	At baseline, 1 h after the endurance exercise session, before the resistance exercise session, and 1, 2, and 3 h after the resistance exercise session	1. No change of plasma decorin at 1, 2, and 3 h post resistance exercise compared to baseline.	[94]
A single bout of resistance exercise—bilateral knee extensions: With blood-flow restriction: 1 set of 30 repetitions, followed by 3 sets of 15 repetitions at 30% of one-repetition maximum, with 30 s rest between sets. Without blood-flow restriction: Low-intensity exercise—one set of 30 repetitions and three sets of 15 repetitions at 30% of one-repetition maximum, with 30 s rest between sets. High-intensity exercise—four sets of 7 repetitions at 80% of one-repetition maximum, with 1 min rest between sets.	Men, aged 18–35 years, physically active (1 year of resistance training experience) (*n* = 9)	Before, immediately after, 1 h and 24 h post-exercise	1. Plasma concentration of decorin immediately post-exercise was 11.91% greater than immediately pre-, 1-h post-exercise, and 24-h post-exercise. 2. Low-intensity resistance exercise: pre-exercise 2016.10 ± 1250.94 pg/mL, post-exercise 2195.20 ± 1362.85 pg/mL, 1 h post-exercise 2117.57 ± 1387.60 pg/mL, 24 h post-exercise 1895.50 ± 1182.22 3. Blood-flow restriction resistance exercise: pre-exercise 1901.19 ± 1144.05 pg/mL, post-exercise 2121.24 ± 1189.63 pg/m, 1 h post-exercise 1996.01 ± 1196.53 pg/mL, 24 h post-exercise 1909.76 ± 1187.80 4. High-intensity resistance exercise: pre-exercise 1939.47 ± 1142.65, post-exercise 2237.72 ± 1446.64 pg/mL, 1 h post-exercise 1983.09 ± 1323.87 pg/mL, 24 h post-exercise 1883.66 ± 1090.47 5. No differences in decorin release between interventions	[95]
**Regular (chronic) exercise effects**				
HICT with body mass, 3× per week for 5 weeks. One HICT session consisted of 3 circuits with 2-min breaks between. Each HICT training consisted of 9 exercises with one’s body as a workload performed as follows: jumping jacks, pushups, sit-ups, side plank, squats, plank, running in place, lunges, and push-ups with rotation	Women mean age 40 years, Trained (*n* = 20) and control (*n* = 13).	Before and 1 and 24 h after the first and last session	1. The intervention did not modify the resting plasma decorin concentration	[76]

Abbreviations: 1 RM—one repetition maximum, the maximum amount of weight that a person can lift for one repetition; HICT—high-intensity circuit training; VO2max—the maximum rate of oxygen consumption; y—years old.

**Table 5:** Effects of exercise on BDNF.

Forms of Exercise	Group Characteristics	Blood Samples	Effect of Exercise on the Cytokine Concentration	References
**Acute exercise effects**				
Meta-analyses: Aerobic exercise (15 studies); strength or resistance training (5 studies); strength and endurance training (2 studies); Other (7 studies)	1111 (29 studies) mean age 42.1 years 46.6% of women 14 studies— acute effect	Up to 60 min after exercise	1. Increase in BDNF concentration following a single session of exercise (overall, 1.46-fold increase analyzing 14 studies)	[102]
35 min sessions of physical exercise of moderate intensity compared to cognitive training or mindfulness practice	Men and women, aged 65–85 years, 11 women, 8 men (Total *n* = 19)	Immediately before, immediately after, and at 20 and 60 min after each exercise	1. Only physical exercise produced a significant increase in BDNF serum concentration 2. Physical exercise: before intervention 19.21 ± 1.17 ng/mL, 0 min after 22.72 ± 1.17, 20 min after 21.73 ± 1.2 ng/mL, 60 min after 24.33 ± 1.38 ng/mL 3. Cognitive training: before intervention 20.06 ± 0.95 ng/mL, 0 min after 20.81 ± 1.35, 20 min after 19.89 ± 1.35 ng/mL, 60 min after 20.12 ± 1.13 ng/mL 4. Mindfulness practice: before intervention 21.6 ± 1.58 ng/mL, 0 min after 20.41 ± 1.48, 20 min after 21.76 ± 1.05 ng/mL, 60 min after 20.86 ± 1.74 ng/mL	[103]
30 min physical exercise high or low intensity (cycle ergometer) or relaxing phase	Men and women, aged 18–29 years, 41 women, 40 men, (Total *n* = 81)	At the beginning of the experiment, after the learning phase and after the exercise/relaxing phase	1. Serum BDNF concentration increased after exercising only in the high-intensity exercise group (~15% increase)	[104]
Single strength training session in a leg press, knee extensor, and leg curl (4 sets, training session about 35–40 min). Trained group (6 months prior training) and untrained group	Men, mean age 26.6 years, mean BMI 24.1 (*n* = 20)	Immediately before, immediately after, 2 and 24 h after the training	1. The BDNF serum concentration increased only in the trained group immediately after the end of exercise (~85% increase) and not 2 or 24 h after the training	[105]
12-week CrossFit program (high-intensity interval training). Sixty-minute workouts twice a week.	Men and women, mean age 25.6 years, 7 male, 5 women (Total *n* = 12)	At rest and 15 min after Wingate test and 15 min after progressive test—before and after the intervention (12-week CrossFit program)	1. Before intervention—increase in serum BDNF directly after the Wingate test and progressive test (~10% in men, ~30% in women for Wingate test; ~40% in men, ~80% in women after progressive test) 2. After 12-week CrossFit program—decrease in serum BDNF directly after the Wingate test and progressive test (~15% in men after Wingate test, ~20% in men, ~10% in women after progressive test)	[106]
30 min of aerobic exercise at 60% of individual VO2max. Subjects divided into higher fitness group (VO2max > 75 percentile) and lower fitness group (VO2max < 45 percentile)	Men, Aged 18–28 years, (*n* = 60)	Pre- and post-exercise	1. Serum BDNF concentration was higher post-exercise than in the pre-exercise in the higher fitness group (86.09 ± 68.14 pg/mL vs. 53.06 ± 34.65 pg/mL) 2. No changes in the lower fitness group (an increase nearly reaching significance 57.07 ± 71.42 pg/mL vs. 39.78 ± 38.60 pg/mL)	[107]
High-intensity, low-volume strength training (at 90% 1 RM) (HI) or a high-volume, moderate-intensity strength training (at 70% 1 RM) (HV). Training program—4 times a week for 7 weeks	Men, mean age 23.5 years, Active, strength-trained (*n* = 20)	During the first training session of week 1 and week 7—at baseline, immediately post-exercise, 30 min post-exercise, and 60 min post-exercise.	1. Elevations in plasma BDNF concentrations from baseline, immediately, and 60 min post-exercise in both HI and HV combined before and after the intervention (training program) 2. Before intervention: High-intensity, low-volume post-exercise (~100% increase immediately post-exercise and ~100% increase 60 min post-exercise) 3. After intervention: High-intensity, low-volume post-exercise (~50% increase immediately post-exercise and ~100% increase 60 min post-exercise) 4. Before intervention: Low-intensity, high-volume post-exercise (~180% increase immediately post-exercise and ~200% increase 60 min post-exercise) 5. After intervention: Low-intensity, high-volume post-exercise (~50% increase immediately post-exercise and ~75% increase 60 min post-exercise)	[108]
HIIT (High-intensity interval training) protocol—intervals of 1 min (90% maximal workload) alternating with 1 min rest at 60 W for a total of 20 min CON (continuous exercise) protocol cycle ergometer at the same intensity for 20 min (at 70% of maximal workload)	Experiment 1 7 Men (*n* = 7) Experiment 2 26 Men aged 22–35 years, (*n* = 26)	30 min before exercise, during exercise (0, 6, 10, 14, 18, and 20 min), 20 min after exercise (experiment 1) 30 min before exercise, the start of exercise, the end of exercise (experiment 2)	1. Serum BDNF concentration increased gradually during exercise in both protocols, reaching maximum concentrations toward the end of the exercise. (experiment 1)(CON—increase ~30% and HIT—increase ~45%) 2. BDNF serum concentration returned quickly to baseline after exercise—the measurement 20 min post-exercise was not significantly different from that at rest levels (experiment 1) 3. Both exercise protocols—an increase of serum BDNF concentration compared with a rest condition, HIT reached higher BDNF serum concentration than CON (experiment 2)	[109]
A 6-week supervised physical training program	Men, mean age 23.8 years, (*n* = 34)	Before and after the 6-week exercise intervention	1. Decreased exercise-induced serum BDNF concentration after intervention in the exercise group; nonsignificant changes in the control group 2. Before training intervention: in control group baseline 8.1 ± 3.6 ng/mL, after exercise 12.3 ± 3.9 ng/mL and in the exercise group baseline 12.4 ± 5.8 ng/mL, after exercise 16.7 ± 7.7 ng/mL 3. After training intervention: in control group baseline 8.1 ± 2.8 ng/mL, after exercise 14.8 ± 5.9 ng/mL and in the exercise group baseline 10.1 ± 4.2 ng/mL, after exercise 11.1 ± 4.4 ng/mL	[110]
Three sessions performed on three separate days randomly for all participants at low (<60% VO2max—90% of VT1), moderate (60–75% VO2max—the midpoint between VT1 and VT2), and high (>90% VO2max—the midpoint between VT2 and Wmax) intensities until exhaustion or for up to 60 min	38 Men mean age 28.8 years, (*n*= 38)	pre- (rest) and immediately post-exercise session	1. Increase of BDNF serum concentration after exercise in all the intensity groups. 2. Individuals with lower physical fitness (<49.7 mL/kg/min) exhibited greater BDNF changes, mainly after high-intensity with a short-time, when compared with well-trained individuals with better physical fitness 3. Low intensity: pre 33440.85 ± 6229.58 pg/mL, post 34,900.17 ± 6908.31 pg/mL 4. Moderate intensity 28,169.05 ± 4674.63 pg/mL, post 32,793.15 ± 5198.64 pg/mL 5. High intensity 26,673.73 ± 4896.58 pg/mL, post 43,542.48 ± 6774.00 pg/mL	[111]
**Regular (chronic) exercise effects**				
Meta-analyses: Aerobic exercise (15 studies); Strength or resistance training (5 studies); Strength and endurance training (2 studies); Other (7 studies)	1111 (29 studies) mean age 42.1 years 46.6% women	Up to 60 min after exercise	1. Regular exercise (range 3–24 weeks) caused a more significant increase of BDNF concentration after a session of exercise (overall 1.58-fold increase analyzing 8 studies) 2. Regular exercise (range 3 weeks–2 years) caused a greater increase (but smaller than the 3–24 weeks range) increase of BDNF concentration after a session of exercise (overall 1.28-fold increase analyzing 13 studies)	[102]
Low, moderate, and high-intensity exercises (40%, 55%, 70% VO2max) on the treadmill four times a week for 12 weeks, 200 kcal burn in each session. Control group—stretching.	Men, mean age 15 years, (*n* = 40)	Before intervention and after 12 weeks	1. Increase in serum BDNF concentration at rest compared to pre-intervention in the moderate-intensity exercise and high-intensity exercise groups. 2. No changes in the low-intensity exercise group or stretching group. 3. Low intensity exercise group: pre 24.79 ± 25.77 ng/mL, post 25.05 ± 21.47 ng/mL 4. Moderate intensity exercise group: pre 25.90 ± 26.59 ng/mL, post 27.71 ± 25.86 ng/mL 5. High intensity exercise group 25.24 ± 34.17 ng/mL, post 30.09 ± 48.00 ng/mL 6. Stretching group pre 23.96 ± 20.93 ng/mL, post 24.50 ± 22.04 ng/mL	[112]
12-week CrossFit program (high-intensity interval training). Sixty-minute workouts twice a week.	Men and women, mean age 25.6 years, 7 male, 5 women (Total *n* = 12)	At rest and 15 min after Wingate test and 15 min after progressive test—before and after the intervention (12-week CrossFit program)	1. Resting serum BDNF concentration increased after CrossFit training in men and women. (increase ~50% in women and ~50% in men)	[106]
High-intensity, low-volume strength training (at 90% 1 RM) (HI) or a high-volume, moderate-intensity strength training (at 70% 1 RM) (HV). Training program—4 times a week for 7 weeks	Men, mean age 23.5 years, Active, strength- trained (*n* = 20)	During the first training session of week 1 and week 7—at baseline, immediately post-exercise, 30 min post-exercise, and 60 min post-exercise.	1. A training program of 7-week strength exercises increased the plasma BDNF response to exercise irrespective of exercise severity protocol 2. No change of resting BDNF plasma concentration was reported 3. Before intervention: High-intensity, low-volume post-exercise (~100% increase immediately post-exercise and ~100% increase 60 min post-exercise) 4. After intervention: High-intensity, low-volume post-exercise (~50% increase immediately post-exercise and ~100% increase 60 min post-exercise) 5. Before intervention: Low-intensity, high-volume post-exercise (~180% increase immediately post-exercise and ~200% increase 60 min post-exercise) 6. After intervention: Low-intensity, high-volume post-exercise (~50% increase immediately post-exercise and ~75% increase 60 min post-exercise)	[108]
A 6-week supervised physical training program	Men, mean age 23.8 years, (*n* = 34)	Before and after the 6-week exercise intervention	1. No change in baseline serum BDNF concentration after a 6-week training program. 2. No change in baseline BDNF in the control group	[110]
Regular endurance exercise: 16 weeks of taekwondo training 5 × 60 min per week	Men and women, mean age 12.6 years, overweight and obese adolescents Exercising group (*n* = 10) Control group (*n* = 10) 7 men, 3 women (total *n* = 20)	Before and after 16 weeks of training	1. Increase in serum BDNF concentration after a 16-week intervention 2. Exercise group pre 25.41 ± 5.36 ng/mL, post 29.52 ± 5.83 ng/mL 3. Control group pre 26.58 ± 6.10 ng/mL, post 27.68 ± 6.50 ng/mL	[113]

Abbreviations: BDNF—brain-derived neurotrophic factor; 1 RM—one repetition maximum, the maximum amount of weight that a person can lift for one repetition; VO2max—the maximum rate of oxygen consumption; VT1—ventilatory threshold 1, VT2—ventilatory threshold 2; CON—continuous exercise, HIT—high-intensity training, HI—high intensity, HV—high volume, HIIT—high-intensity interval training; y—years old.

**Table 6:** Effect of exercise on FGF21.

Forms of Exercise	Group Characteristics	Blood Samples	Effect of Exercise on the Cytokine Concentration	References
**Acute exercise effects**				
A single session of treadmill exercise with increasing intensity. Mean duration of one trial 14.2 min. Regular endurance training: 9 supervised trials within 14 days.	Women, aged 18–35 years, sedentary (*n* = 60)	Before exercise, 1 h post, 4 h post-exercise. After 2 weeks of exercise.	No change at any time-point after an acute bout of exercise.	[130]
Two endurance exercise sessions: 30 min treadmill running at 50% VO2max (1st trial) and 80% VO2max (2nd trial). Trials separated by 3 days.	Men, mean age 22.1 years, non-athletic (50% VO2max *n* = 13 80% VO2max *n* = 8)	Before exercise, immediately post, 1 h post-exercise.	FGF21 serum concentration increased 1 h after exercise: ~3 fold after mild-intensity (50% VO2max) and ~5 fold after high-intensity (80% VO2max) training. Concentration after high-intensity training was significantly higher than after mild-intensity. No change immediately post-exercise.	[126]
Three sessions: Moderate-intensity endurance exercise: treadmill run at 55% VO2max to energy expenditure 600 kcal (mean 57 min). High-intensity endurance exercise: treadmill run at 75% VO2max to energy expenditure 600 kcal (mean 42 min). Control—rest. Participants performed all training sessions with at least a 5-day interval.	Men, mean age 26 years, (*n* = 10)	Blood samples before exercise, immediately after, 1 h, 2 h, 4 h, 7 h after the exercise	Serum FGF21 concentrations increased up to 4 h post-exercise compared to control. More significant increases were observed at 1 h, 2 h, and 4 h after high-intensity exercise vs. moderate-intensity training. Area under FGF21 concentration versus time curve (baseline to 2 h post-exercise [pg/mL]) during control: 144 ± 124, mild intensity training: 230 ± 156 and high intensity training: 334 ± 249	[92]
60 min cycling at 75% VO2max	Men, mean age 23.7 years, sedentary (*n* = 19)	Before exercise, immediately post-exercise.	FGF21 serum concentration increased immediately post-exercise.	[127]
A single session of endurance exercise: 60 min of treadmill running at 60% VO2max	Men, mean age 36 years, sedentary or moderately active (*n* = 11)	Before exercise, 1 h, 1.5 h, 2.75 h, 4 h and 7 h post-exercise.	FGF21 serum concentration increased at 1 h, 1.5 h, and 4 h post-exercise compared to baseline. Peak values: ~2-fold increase at 1.5 h post-exercise compared to baseline.	[91]
A single endurance exercise session: 30 min cycling at 70% VO2max preceded with carbohydrate intake (180 kcal).	Men, aged 18–22 years, (*n* = 7) Men, aged 62–69 years, (*n* = 8)	Before exercise, immediately after, 30 min post, 1 h post, 3 h post, 24 h post-exercise.	No change at 30 min, 1 h, and 3 h post-exercise. A significant decrease in serum FGF21 concentration 24 h post-exercise compared to baseline, immediately post 30 min and 1 h post-exercise	[131]
Endurance exercise: 1 h cycling at 70% VO2max. Resistance exercise: 5 sets of high-volume exercises involving major muscle parts within 1 h. Participants completed both modes of exercise in a cross-over design. Trials separated by 6–12 days.	Men, mean age 24 years, recreationally active (*n* = 10)	Before exercise, immediately after, 15 min, 30 min, 1 h, 90 min, 2 h, 3 h post-exercise.	Endurance exercise: a significant increase in FGF21 serum concentration starting at 15 min post-exercise with peak at 1 h post-exercise (~ 3-fold increase) until 2 h post-exercise. Resistance exercise: no change in serum FGF21 concentration.	[128]
1st day: 2 min blocks of cycling alternating between 90 and 50% of maximal workload 2nd day: 90 min cycling at 50% of maximal workload Retest- the same protocol after 1 week	Men, mean age 27 years, well-trained (*n* = 11)	Before exercise, immediately after exercise performed on the 2nd day, 1 h post, 24 h post Retest- the same protocol after 1 week	Test: FGF21 increased over 2-fold 1 h after the 2nd day exercise. Retest: no change in FGF21 serum concentration.	[129]
3 sessions of endurance and resistance exercise separated by 7-day rest: High-intensity interval training 1: 2 sets of 6 × 30 s treadmill running at Vmax eliciting VO2max, with 90 s of active recovery (50% of Vmax) between bouts and 4 min of passive recovery between sets. High-intensity interval training 2: 5 × 4 min treadmill running at 90% Vmax, with 4 min of active recovery (50% of Vmax) between bouts. Resistance Training session: 7 types of exercises targeting main muscle groups. 4 sets of 8–10 repetitions at 70–75% of one-repetition maximum with 60–90 s of rest between exercises and for 4 min between sets.	Men, mean age 23 years, sedentary (*n* = 17)	Before exercise, immediately after, 1 h, 3 h, 24 h, 48 h, and 72 h after each training session.	Serum FGF21 increased after all training types. High-intensity interval training induced an increase immediately and 3 h after the exercise, whereas resistance training after 48 h. High-intensity interval training 1: less than 1.5-fold increase. High-intensity interval training 2: over 2-fold increase. Resistance Training: over 2-fold increase.	[79]
A single session of resistance exercise: maximal single-leg eccentric contractions—3 sets of 25 repetitions separated with 5 min rest.	Men, mean age 25.0 years, physically active (*n* = 8)	Before exercise, after each exercise set, every 20 min during 3 h recovery.	No significant changes in FGF21 serum concentration at all post-exercise time points compared to baseline.	[134]
**Regular (chronic) exercise effects**				
A single session of treadmill exercise with increasing intensity. Mean duration of one trial 14.2 min. Regular endurance training: 9 supervised trials within 14 days.	Women, aged 18–35 years, sedentary (*n* = 60)	Before exercise, 1 h post, 4 h post-exercise. After 2 weeks of exercise.	FGF21 serum concentration increased over 1.5-fold compared to baseline. Baseline: 276.8 ng/L. After 2 weeks: 460.8 ng/L.	[130]
5 weeks of supervised endurance exercise. 3 sessions of cycling each week with increasing intensity (60 VO2max, 70%, and 75%) and duration (30 min, 45 min). 5 weeks without regular physical activity. Participants were randomized to the exercising or resting group, then changed in a cross-over design.	Men, mean age 69.6 years, (*n* = 27)	Before exercise, 5 weeks post, 10 weeks post-exercise.	Serum FGF21 concentration decreased after 5 weeks of training. Pre-exercise: 248.1 ± 88.5 pg/mL Post-exercise: 218.5 ± 94.2 pg/mL	[133]

Abbreviations: FGF21—fibroblast growth factor 21; VO2max—the maximum rate of oxygen consumption; y—years old.

**Table 7:** Effects of exercise on IL-15.

Forms of Exercise	Group Characteristics	Blood Samples	Effect of Exercise on the Cytokine Concentration	References
**Acute exercise effects**				
A single session of resistance exercise: 4 sets of 10 repetitions of a back squat exercise at 70% of one-repetition maximum, using either traditional set configurations (4 × 10 with 180 s inter-set rest) or cluster sets (4 × (2 × 5) with 30 s intra-set rest and 150 s inter-set rest). Participants performed both training modes with a 7-day interval.	Men, mean age 27 years, resistance-trained (*n* = 10)	Blood samples before, immediately after, 30 min, 60 min, 24 h, and 48 h post-exercise	IL-15 serum concentration increased immediately post-exercise by 20–30% after both types of set configurations, then it returned to baseline values.	[144]
A single session of resistance exercise: 4 sets of 8–15 repetitions of bilateral leg press and knee extension at 75% of one-repetition maximum	Men, mean age 24.9 years, resistance-trained (*n* = 14)	Blood samples: before, mid-exercise, immediately after, 0.3 h, 1 h, 2 h, 4 h, 24 h post-exercise.	IL-15 serum concentration: increase by ~3.5-fold immediately after exercise; remained elevated until 24 h post-exercise.	[145]
An endurance exercise session: 90 min cycling at 70% VO2max followed by a resistance exercise session: 5 sets of 8 repetitions of bilateral leg press and leg extensions at 80% of one-repetition maximum with 2 min rest between sets and 4 h rest and meal between both exercise sessions. Participants performed the intervention twice either with carbohydrate or fat meal.	Men, mean age 21.2 years, recreationally active (*n* = 13)	Blood samples: before, 1 h after endurance exercise, 1 h after the meal, 1 h, 2 h and 3 h after resistance exercise.	IL-15 serum concentration increased by 15–25% 1 h after resistance exercise irrespective of meal type. No changes 1 h post endurance exercise.	[94]
A single session of resistance exercise—bilateral knee extensions: With blood-flow restriction: 1 set of 30 repetitions, followed by three sets of 15 repetitions at 30% of one-repetition maximum, with 30 s rest between sets. Without blood-flow restriction: Low-intensity exercise—1 set of 30 repetitions and 3 sets of 15 repetitions at 30% of one-repetition maximum, with 30 s rest between sets. High-intensity exercise—4 sets of 7 repetitions at 80% of one-repetition maximum, with 1 min rest between sets.	Men, aged 18–35 years, physically active (1 year of resistance training experience) (*n* = 9)	Blood samples before, immediately after, 1 h and 24 h post-exercise	No changes in serum IL-15 concentration.	[95]
Single resistance training session: leg press, knee extension, leg curl 4 sets of 8–10 repetitions of each exercise at 65% one-repetition maximum, completed within 35–40 min.	Men, mean age 26.6 years, Trained group (*n* = 10) Untrained group (*n* = 10)	Blood samples before, immediately after, 2 h and 24 h after the training session	No changes in serum IL-15 after a single session of resistance exercise in both groups.	[105]
Three sessions of endurance and resistance exercise separated by 7 days rest: High-intensity interval training 1: 2 sets of 6 × 30 s treadmill running at Vmax eliciting VO2max, with 90 s of active recovery (50% of Vmax) between bouts and 4 min of passive recovery between sets. High-intensity interval training 2: 5 × 4 min treadmill running at 90% Vmax, with 4 min of active recovery (50% of Vmax) between bouts. Resistance Training session: 7 types of exercises targeting main muscle groups. 4 sets of 8–10 repetitions at 70–75% of one-repetition maximum with 60–90 s of rest between exercises and for 4 min between sets.	Men, mean age 23 years, sedentary (*n* = 17)	Blood samples before, immediately after, 1 h, 3 h, 24 h, 48 h, and 72 h after each training session.	No changes in serum IL-15 after a single session of both resistance and endurance exercise.	[79]
Two sessions of eccentric and concentric emphasized resistance exercise with a 4-day interval. Each session comprised three sets of 8–10 repetitions of 7 exercises involving all major muscle groups (squat, chest press, seated row, leg extensions, triceps extensions, arm curl, shoulder press, and hamstring curl) at 70–80% of one-repetition maximum for concentric and 90–100% of one-repetition maximum for eccentric emphasized exercise.	Men, non-athletes, mean age 20.8 years, (*n* = 14) Men, athletes, mean age 24.1 years., (*n* = 14)	Blood samples before and immediately after (1–5 min) each training session.	An increase in IL-15 concentration after ECC (1.43 ± 0.17 vs. 1.62 ± 0.28 pg/mL) and CON (1.79 ± 0.6 vs. 2.16 ± 0.6 pg/mL) resistance exercise in non-athletes. In athletes, IL-15 serum concentration increased significantly only after ECC resistance exercise (1.72 ± 0.4 vs. 2.46 ± 1.3 pg/mL), which was noted to be the highest degree of change in IL-15 in all subjects. The increase after the CON was insignificant in this group.	[147]
Resistance exercise—static: holding a rod below the knees until exhaustion. Endurance exercise: 5 min cycling with the power adjusted for weight followed by 3-min rest and 5-min cycling with the power adjusted to the heart rate measured at the end of the first load.	Men, aged 18–23 years, weightlifting group (elite strength-trained athletes; *n* = 10) track and field group (elite endurance-trained athletes; *n* = 10) control group 1 and control group 2 included 10 untrained volunteers	Blood samples before, immediately after, 30 min post-exercise.	Static resistance exercise increased IL-15 concentration by ∼50% in athletes only. Endurance exercise had no impact on IL-15 in both athletes and untrained individuals.	[148]
Wingate test (30 s trial on a cycle ergometer with pedaling rate of 100 RPM). 4 conditions: normoxia (4 min breathing with room air before the test) after placebo or antioxidant intake and hypoxia (4 min breathing with the gas mixture with reduced oxygen concentration before the test) after placebo or antioxidant intake.	Men mean age 25.2 years, physically active (*n* = 9)	Blood samples and muscle biopsies before, immediately after, 30 min, and 120 min post-exercise.	IL-15 muscle protein content increased significantly after the exercise.	[149]
A single session of strenuous endurance exercise: 35 km trail run with total climb of 940 m completed within 6 h.	Women (*n* = 11) and Men (*n* = 26) (27 included in IL-15 concentration analysis) mean age 38.97 years	Blood samples before and after the run.	IL-15 serum concentration increased by 2.22-fold after the run compared to the baseline.	[150]
HICT with body mass, 3× per week for 5 weeks. One HICT session: 3 circuits of 9 exercises (jumping jacks, pushups, sit-ups, side plank, squats, plank, running in place, lunges, and push-ups) with one’s body as a workload with 2 min rest between circuits. The control group performed HICT twice at the baseline and after 5 weeks	Women Exercising group (*n* = 20) Control group (*n* = 13). Age subdivision: young (*n* = 11) and middle-aged (*n* = 9)	Blood samples. Before, 1 h and 24 h after the first and last session.	First HICT session induced a decrease in serum IL-15 by 5–25% in both groups (training and control).	[76]
**Regular (chronic) exercise effects**				
12-week resistance training protocol: 60 min per day 3 days per week Three sets of 12 repetitions of 7 exercises (squat, lunge, chest press, vertical fly, lat pull down, long pull, crunch) at 55–65% of one-repetition maximum	Women, premenopausal, mean age 47.5 years, overweight (*n* = 15) Women, postmenopausal, mean age 57.8 years, normal-weight (*n* = 20)	Blood samples before and after complete training protocol (12 weeks).	A significant increase in IL-15 concentration after 12 weeks of regular resistance training. Premenopausal: 34.94 ± 2.76 vs. 25.90 ± 3.42 Postmenopausal: 33.54 ± 4.48 vs. 26.37 ± 3.24	[146]
HICT with body mass, 3× per week for 5 weeks. One HICT session: 3 circuits of 9 exercises (jumping jacks, pushups, sit-ups, side plank, squats, plank, running in place, lunges, and push-ups) with one’s body as a workload with 2 min rest between circuits. The control group performed HICT twice at the baseline and after 5 weeks	Women, Exercising group (*n* = 20) Control group (*n* = 13). Age subdivision: young (*n* = 11) and middle-aged (*n* = 9)	Blood samples. Before, 1 h and 24 h after the first and last session.	Last HICT session: increase by~10% in serum IL-15 only in the training group.	[76]
12 weeks of regular, supervised endurance exercise: bench step exercise at moderate intensity (lactate threshold) 3× per day, 10–20 min each	Women, aged 65–85 years, Exercising group (*n* = 31) Control group (*n* = 31)	Blood samples before and after 12 weeks of training.	No significant changes in serum IL-15 after 12 weeks of regular exercise.	[151]
Regular endurance exercise: 16 weeks of taekwondo training 5 × 60 min per week	Men and women, mean age 12.6 years, overweight and obese adolescents Exercising group (*n* = 10) Control group (*n* = 10) 7 men, 3 women (total *n* = 20)	Blood samples before and after 16 weeks of training	No significant IL-15 serum concentration changes after 16 weeks of regular exercise both within the exercising group and between groups.	[113]

Abbreviations: IL-15—interleukin 15; VO2max—the maximum rate of oxygen consumption; Vmax—maximal speed; ECC—eccentric emphasized exercise group; CON—concentric emphasized group, HICT—high-intensity circuit training; y—years old.
